# Cholesterol Depletion Disorganizes Oocyte Membrane Rafts Altering Mouse Fertilization

**DOI:** 10.1371/journal.pone.0062919

**Published:** 2013-04-25

**Authors:** Jorgelina Buschiazzo, Come Ialy-Radio, Jana Auer, Jean-Philippe Wolf, Catherine Serres, Brigitte Lefèvre, Ahmed Ziyyat

**Affiliations:** 1 INSERM U1016, Institut Cochin, Université Paris Descartes, 24 rue du Faubourg Saint-Jacques, F75014 Paris, France; 2 Instituto de Investigaciones Bioquímicas de Bahía Blanca (UNS-CONICET), Bahía Blanca, Argentina; 3 INSERM U1016, Institut Cochin, Université Paris Descartes, 24 rue du Faubourg Saint-Jacques. F75014 Paris, France; 4 Service d’Histologie Embryologie Biologie de la Reproduction Hôpital Cochin, AP-HP, F75014 Paris, France; Institut Jacques Monod, France

## Abstract

Drastic membrane reorganization occurs when mammalian sperm binds to and fuses with the oocyte membrane. Two oocyte protein families are essential for fertilization, tetraspanins and glycosylphosphatidylinositol-anchored proteins. The firsts are associated to tetraspanin-enriched microdomains and the seconds to lipid rafts. Here we report membrane raft involvement in mouse fertilization assessed by cholesterol modulation using methyl-β-cyclodextrin. Cholesterol removal induced: (1) a decrease of the fertilization rate and index; and (2) a delay in the extrusion of the second polar body. Cholesterol repletion recovered the fertilization ability of cholesterol-depleted oocytes, indicating reversibility of these effects. In vivo time-lapse analyses using fluorescent cholesterol permitted to identify the time-point at which the probe is mainly located at the plasma membrane enabling the estimation of the extent of the cholesterol depletion. We confirmed that the mouse oocyte is rich in rafts according to the presence of the raft marker lipid, ganglioside GM1 on the membrane of living oocytes and we identified the coexistence of two types of microdomains, planar rafts and caveolae-like structures, by terms of two differential rafts markers, flotillin-2 and caveolin-1, respectively. Moreover, this is the first report that shows characteristic caveolae-like invaginations in the mouse oocyte identified by electron microscopy. Raft disruption by cholesterol depletion disturbed the subcellular localization of the signal molecule c-Src and the inhibition of Src kinase proteins prevented second polar body extrusion, consistent with a role of Src-related kinases in fertilization via signaling complexes. Our data highlight the functional importance of intact membrane rafts for mouse fertilization and its dependence on cholesterol.

## Introduction

At the time of fertilization, when a spermatozoon encounters an oocyte, it first binds to its membrane and then, both membranes fuse together. Drastic oocyte membrane reorganization occurs (for review [1]). Concerning the proteins of the oocyte membrane clearly involved in the process of gametes adhesion/fusion, one is the tetraspanin Cd9 [2], [3], [4], the other is, at least, one but still unknown, glycosylphosphatidylinositol-anchored protein (GPI-anchored protein) [5], [6]. We have already hypothesized on the links between these two proteins and in particular on the membrane reorganization at the time of gametes adhesion/fusion [7]. The basic structure of cell membranes is the lipid bilayer, composed of two apposing leaflets, forming a two-dimensional liquid with fascinating properties designed to perform the functions cells require [8]. To coordinate these functions, the bilayer has evolved the propensity to segregate its constituents laterally to form specialized functional microdomains permitting membrane subcompartmentalization and the formation of signaling platforms [9]. Among these microdomains are the tetraspanin enriched microdomains (TEM), caveolae, and lipid rafts. These last ones combine the potential for sphingolipid-cholesterol self-assembly with protein specificity to focus and regulate membrane bioactivity [8]. Moreover, one of the lipids known to promote raft association is the GPI anchor, and as said above at least one protein anchored to GPI is essential in gamete adhesion/fusion [5], [6]. Another lipid constituting the rafts is the ganglioside GM1, which is expressed on the mouse oocyte and cleaving embryos [10] showing a differential distribution with respect to monosialylGb5Cer-enriched membrane rafts in preimplantation embryos [11]. However, little data have been published on the comportment and role of membrane rafts during mammalian fertilization, neither on their associated proteins such as flotillins and caveolins [12], [13] or tyrosine kinases involved in oocyte activation (for review [14]). Thus, the aim of this work was to study membrane raft domains to characterize their components and evaluate their functional significance in relation to mouse oocyte fertilization.

## Materials and Methods

### 1- Gamete Preparation and in vitro Fertilization

#### Oocyte recovery

This work submitted for ethical evaluation to the “Comité d’Ethique pour l’Expérimentation Animale, Paris Descartes” has been approved and registered under the number CEEA34.BL.006.12.

B6CBA F1 female mice (5–8 week old) purchased from Janvier Laboratories (France) were ovary stimulated with 5 IU PMSG and 5 IU hCG (Intervet, France) 48 hours later. Twelve to 14 hours after hCG injection, female were sacrificed by cervical dislocation. Cumulus oophorus were collected by tearing the ampulla’s wall of the oviduct and placed in Ferticult Medium (FertiPro, Belgium) at 37°C under 5% CO_2_ in air under mineral oil (Sigma). When needed, cumulus cells were removed by a brief exposure to hyaluronidase (Sigma) (0.01%) and zona pellucida (ZP) dissolved with acidic Tyrode’s solution (pH 2.5) (Sigma) under visual monitoring. The ZP-free eggs were rapidly washed five times and kept at 37°C under 5% CO_2_ in air for 2 recovery hours.

#### Sperm preparation

Mouse spermatozoa were obtained from the epididymis caudae of B6CBA F1 male mice (8 to 13-week-old) and capacitated at 37°C for 90 minutes in a 500 µl drop of Ferticult Medium with 3% BSA at 37°C under 5% CO_2_ in air under mineral oil.

#### In vitro fertilization

Treated or not treated ZP-free eggs were inseminated with 1×10^5^ capacitated spermatozoa per ml for 1 hour in a 100 µl drop of medium. Then, they were washed and directly mounted in Vectashield medium with DAPI (Vector laboratories, CA, USA) for observation under UV light (Nikon Eclipse E600 microscope). Only oocytes showing at least one fluorescent decondensed sperm head within their cytoplasm were considered fertilized and according to this the fertilization rate (FR) was evaluated. To assess the fertilization index (FI), the number of decondensed sperm heads per oocyte was recorded as well as the extrusion of the second polar body (PB).

### 2- Cholesterol Depletion and Repletion

Methyl-β-cyclodextrin (MβCD; Sigma) was used to deplete cholesterol from ovulated oocytes. A stock solution (1M) in Ferticult medium was stored at 4°C in a glass tube. The stock solution was vortexed 30 minutes at room temperature (RT) before preparing working solutions (5, 15 and 30 mM). After the recovery period, ZP-free oocytes, were treated with MβCD during 30 minutes at 37°C, and then washed in Ferticult medium and inseminated or assessed for fluorescence staining. Only those oocytes that survived MβCD treatment were inseminated or selected for fluorescence staining.

To evaluate the reversibility of cholesterol removal, cholesterol repletion was performed just after washing depleted oocytes. Oocytes were bathed during 30 minutes at 37°C in MβCD/Cholesterol (molar ratio 8∶1) in Ferticult prepared according to Christian *et al.* [15]. Briefly, cholesterol (Sigma) in chloroform:methanol 1∶1 (v:v) was completely dried under a stream of nitrogen. An MβCD aqueous solution at the adequate concentration was subsequently added to the dried material. The mixture was clarified by vigorous mixing and incubated in a rotating water bath at 37°C overnight.

### 3- Sequestration of Cholesterol with Nystatin

Nystatin dihydrate (Sigma) was used to disrupt membrane rafts. A stock solution (5 mg/ml) in DMSO was aliquoted in dark tubes protected from light and stored at −20°C. After the recovery period, ZP-free oocytes were treated with nystatin in Ferticult medium (200 µg/ml) during 1 hour at 37°C, and then washed and inseminated. To exclude for any effects of this solvent the control cells were incubated in the same dilution of DMSO. Nystatin at this concentration did not compromise cell viability.

### 4- Fluorescence Staining of Mouse Oocytes

**Cholesterol imaging in living oocytes.** A stock solution (5 mM) of BODIPY-Cholesterol (BPY-Chol; Avanti Polar Lipids) was prepared in ethanol and stored in a dark glass tube under nitrogen at −20°C. Working solutions (1 µM) were obtained diluting the stock in M2 medium (amount of ethanol less than 1%). Pulse-labeling was performed incubating cumulus-free ZP-intact oocytes with the fluorescent lipid probe for 15 minutes at 37°C. To achieve a high and selective plasma membrane labeling, oocytes were immediately washed and subsequently imaged in cold M2 medium to avoid internalization of the lipid probe. On the contrary, to follow the fluorescent cholesterol internalization, cells were imaged at different chase times after labeling and removal of the lipid probe by washing. Quantification of fluorescence intensity was measured outlining regions of interest (ROI) using ImageJ software. The integrated density and area of a given ROI and the mean fluorescence value of three background selections were measured to calculate the corrected total cell fluorescence (CTCF) [16] according to the formula:

CTCF = Integrated Density - (Area of selected cell × Mean fluorescence of background readings).

#### Detection of molecular raft markers

The presence on the oocyte membrane of three different molecules, caveolin-1, flotillin-2 and the ganglioside GM1, known to participate in membrane rafts constitution was verified.

For the two proteins, the monoclonal antibodies used were anti-flotillin-2 (clone B-6, Santa Cruz Biotechnology Inc.) and anti-caveolin-1 (clone 2297, BD Transduction Laboratories). The secondary antibody was a goat-anti-mouse-Alexa Fluor 488 (AF^488^, Invitrogen). Immunodetection was carried out on cumulus- and ZP-free oocytes fixed in 2% PFA diluted in PBS 1% BSA for 20 minutes at RT. For caveolin-1, oocytes were permeabilized in PBS supplemented with 1% BSA and 0.1% Triton during 15 minutes at RT. They were then incubated in a blocking solution (PBS containing 10% goat serum) during 1 hour at RT and with the primary antibody (1∶50; anti-cav-1 or anti-flot-2) for 1 hour at RT and then, with the secondary antibody (1∶200; goat anti-mouse AF^488^) for 1 hour at RT. Controls were prepared by omitting the primary antibody. The oocytes were washed in PBS 1% BSA and directly mounted in Vectashield/DAPI for observation under UV light (Nikon Eclipse E600 microscope).

The glycosphingolipid GM1 was detected on living cumulus-free ovulated oocytes by using the fluorescent-labeled cholera toxin B subunit (CTB-AF^488^, Molecular Probes), which binds specifically to the ganglioside. Oocytes were incubated at 37°C for 10 minutes in M2 medium (Sigma) supplemented with CTB-AF^488^ (20 µg/ml), mounted in cold M2 medium and transferred on ice to the microscope to avoid rapid internalization of the toxin-GM1.

Taking into account that membrane rafts are associated to Src-kinases, we verified on cumulus-free ovulated, fixed and permeabilized oocytes the presence of the tyrosine kinase Src by immunofluorescence, using the monoclonal antibody anti-c-Src (clone H-12, Santa Cruz Biotechnology Inc.). Immunodetection was carried out on oocytes fixed in 2% PFA diluted in PBS 1% BSA for 20 minutes at RT and permeabilized in PBS supplemented with 1% BSA and 0.1% Triton during 15 minutes at RT. Oocytes were then incubated in a blocking solution (PBS containing 10% goat serum) during 1 hour at RT and subsequently incubated with the primary antibody (1∶50; anti-c-Src) for 1 hour at 4°C. Incubation with the secondary antibody (1∶200; goat anti-mouse AF^488^) was performed for 1 hour at RT. Controls were prepared by omitting the primary antibody. Oocytes were washed in PBS 1% BSA and directly mounted in Vectashield/DAPI for observation under UV light. To evaluate the effect of cholesterol depletion on c-Src localization, oocytes were pretreated with 15 mM MβCD as indicated above.

#### Detection of the non-raft protein Cd9

Expression levels of a non-raft protein tetraspanin Cd9 was evaluated in ovulated oocytes by immunofluorescence after 30 minutes treatment with MβCD 15 mM compared to non-treated oocytes. Oocytes were incubated with anti-Cd9 (1∶50; KMC8, BD Pharmingen, USA) for 45 minutes at RT. Incubation with the secondary antibody (1∶200; goat anti-mouse AF^488^) was performed for 45 minutes at RT. The oocytes were washed in PBS 1% BSA and directly mounted in Vectashield/DAPI for observation under UV light (Nikon Eclipse E600 microscope).

### 5- Western Blot Analysis

Whole oocyte proteins were resolved by SDS-PAGE. Proteins in the gel were transferred to a polyvinylidene difluoride (PVDF) membrane (Hybond-P; GE Healthcare Ltd., U.K) by using a Mini-Trans-Blot electrophoretic transfer cell (Bio-Rad Life Science Group, Hercules, SA) for 1.5 hours. Membranes were blocked for 1hour at 4°C with TBS buffer (20 mM Tris-HCl, pH 7.5, 150 mM NaCl) supplemented with 5% nonfat dry milk. Immunoblotting detection was performed using a 1∶1000 dilution of anti-flotillin-2 (clone B-6, sc-28320, Santa-Cruz Biotechnology Inc.) or anti-caveolin-1 (Clone 2297, BD Transduction Laboratories) at 4°C overnight. Incubation with the secondary antibody was performed at RT for 45 minutes using a 1∶3000 dilution of rabbit anti-mouse (Dako A/S, DK 2600 Glostrup) or a 1∶30000 dilution of goat anti-rabbit (Vector Laboratories, Inc. Burlingame, CA 94010) conjugated to horseradish peroxidase (HRP) in a TBS buffer supplemented with 0.05% Tween-20 and 1% nonfat dry milk. Immunoreactive bands were detected using the enhanced chemiluminescent HRP Substrate Immobilon Western (Millipore Corporation, MA 01821, USA).

### 6- Src Kinase Inhibition

To verify Src-family protein-tyrosine kinase involvement in the fertilization process, MII oocytes were preincubated in Ferticult medium containing pyrazolopyrimidine 2 (PP2; BD Biosciences, France), a specific Src-family kinase inhibitor, at 0, 10 or 100 µM during 30 minutes at 37°C, and then washed in Ferticult medium and inseminated.

### 7- Ultrastructural Study by Electronic Microscopy

For transmission electron microscopy, cumulus-free oocytes were washed and pre-fixed in a 100 µl drop of 0.25% glutaraldehyde in PBS 1% BSA for 30 minutes and then washed in PBS 1% BSA. After three washes, the oocytes were fixed in 2.5% glutaraldehyde in Sorensen buffer supplemented with 1% BSA for 30 minutes at RT and 1 hour at 4°C. After three washes in Sorensen buffer with 1% BSA the oocytes were post-fixed with 1% osmium tetroxide in 0.1 M phosphate buffer, and then dehydrated in 70%, 90% and 100% ethanol. After 10 minutes in a 1∶2 mixture of epoxy propane and epoxy resin, the oocytes were embedded in gelatin capsules with freshly prepared epoxy resin and polymerized at 60°C for 24 hours. Samples were then mounted into epon blocks and 70 nm thin sections were cut with an ultramicrotome (Reichert ultracut S), stained with uranyl acetate and Reynold’s lead citrate, and observed under a transmission electron microscope (Philips CM10).

### 8- Statistical Analysis

All experiments were realized at least three times. Statistical analysis was carried out using SPSS 15.0 software (Inc., Chicago, IL). Analysis of variance (ANOVA) was used to determine differences among mean values, which were then compared using the *post hoc* tests of multiple comparisons Bonferroni or Fisher’s Least Significant Difference (LSD). Student’s *t* test was used to establish differences between two mean values. Differences were considered significant at *P*<0.05.

## Results

### Effect of Cholesterol Depletion and Repletion on Fertilization

**Figure 1:**
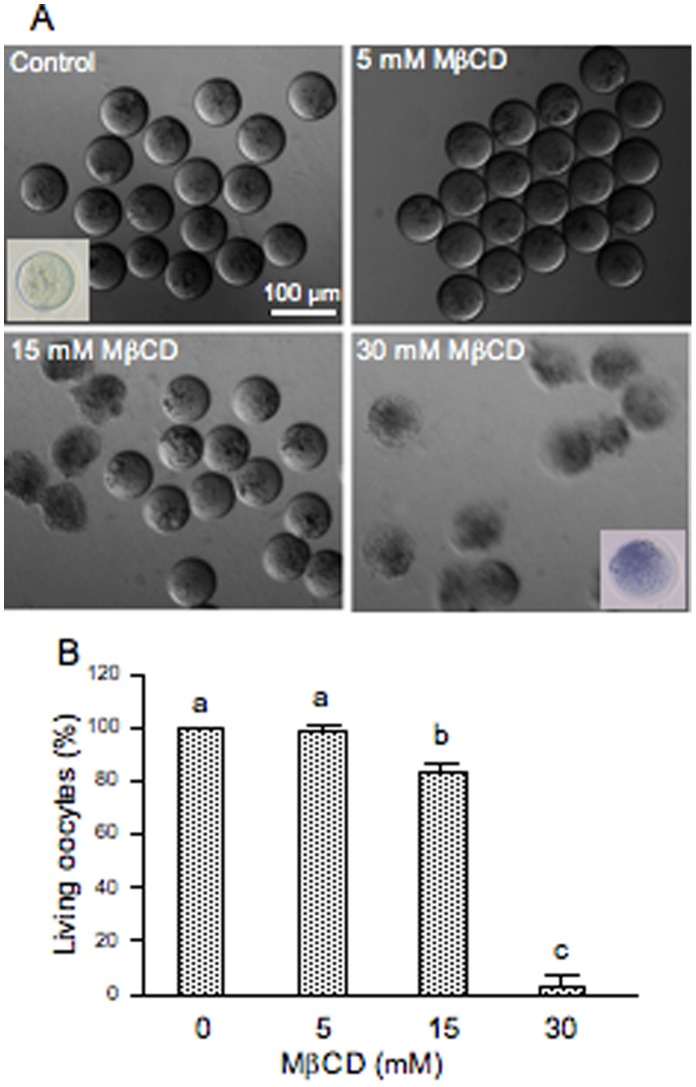
Effect of cholesterol depletion mediated by MβCD on oocyte survival. Zona-free mouse oocytes were incubated with different concentrations of MβCD for 30 min at 37°C. (A) Differential interference contrast micrographs of depleted oocytes after MβCD treatment. Are also illustrated by inserted pictures healthy and dead oocytes demonstrating or not their viability by the trypan blue exclusion test. (B) Percentages of living oocytes after cholesterol depletion. Data represent the mean ± SEM of at least 3 independent experiments from a total of 101 control oocytes, 49 oocytes depleted at 5 mM, 92 oocytes depleted at 15 mM and 29 oocytes depleted at 30 mM of MβCD. Comparison of mean values was performed using Bonferroni test. Different letters (a-c) denote significant differences (*P*<0.05).

**Figure 2:**
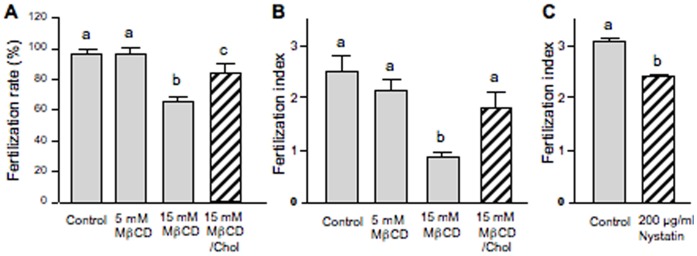
Effect of cholesterol disrupting agents on mouse fertilization. Zona-free mouse oocytes were incubated with either different concentrations of MβCD for 30 min at 37°C to remove cellular cholesterol or 200 µg/ml of Nystatin to sequestrate cholesterol into complexes. Cholesterol repletion was carried out incubating MβCD-treated oocytes with MβCD/Chol complexes. After depletion/repletion and sequestration treatments, oocytes were washed and inseminated. (A) Effect of cholesterol depletion and repletion on the fertilization rateand (B) fertilization index. (C) Effect of Nystatin induced cholesterol sequestration on the fertilization index. Data in A and B represent the mean ± SEM of at least 3 independent experiments from a total of 101 control oocytes, 49 oocytes depleted at 5 mM, 92 oocytes depleted at 15 mM and 52 oocytes depleted/repleted at 15 mM of MβCD. Data in C represent the mean ± SEM of 3 independent experiments from a total of 33 control oocytes and 72 Nystatin-treated oocytes. Comparison of mean values was performed using LSD or Student’s *t* tests. Different letters (a-c) denote significant differences (*P*<0.05).

**Figure 3:**
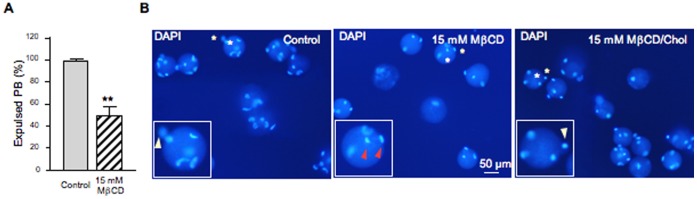
Effect of cholesterol depletion and repletion on polar body extrusion. Zona-free mouse oocytes were incubated with 15 mM of MβCD for 30 min at 37°C to remove cellular cholesterol. Cholesterol repletion was carried out incubating MβCD-treated oocytes with MβCD/Chol complexes. After depletion/repletion treatments, oocytes were washed and inseminated. (A) Percentage of expulsed polar bodies (PB) after fertilization of cholesterol depleted oocytes. (B) Effect of cholesterol depletion and repletion on the extrusion of the second polar body visualized by DAPI staining. Inserts show a zoom of the regions indicated by asterisks. White arrowheads indicate PB and red arrowheads indicate meiosis arrest. Data in A represent the mean ± SEM of 3 independent experiments from a total of 55 control oocytes and 57 oocytes depleted at 15 mM MβCD. Asterisks (**) indicate significant differences with respect to control (*P*<0.01).

The cholesterol-binding drug MβCD was used for oocyte cholesterol modulation in order to evaluate functionality and possible membrane raft involvement in mouse fertilization. If membrane order is essential for fertilization then the possible disruption of membrane microdomains by MβCD should inhibit the sperm-induced response. To deplete cholesterol, ZP-free ovulated oocytes were incubated with different concentrations of MβCD (5–30 mM) and then fertilized with capacitated spermatozoa. Oocytes treated with 15 mM MβCD registered 83% of living oocytes (Fig. 1A,B) whereas none of the oocytes incubated with 30 mM MβCD survived (Fig. 1A,B). As seen on Figure 1A, only healthy oocytes demonstrating their viability by the trypan blue exclusion were used to check their fertilizability. After fertilization, oocytes were mounted and the FR and the FI were recorded. The FR underwent a significant decrease (31%) in oocytes treated with 15 mM MβCD (Fig. 2A). At this concentration, cholesterol removal also decreased by almost 3-fold the FI (Fig. 2B) and significantly inhibited the extrusion of the second PB in 50% of the cases (Fig. 3A). The latter effect was clearly observed in the DAPI-stained image in which segregation of oocyte chromatids was arrested with chromatids still within the ooplasm (Fig. 3B). To assess the reversibility of cholesterol depletion and the specificity of MβCD effects, cholesterol repletion was performed using MβCD/cholesterol complexes. The high affinity of MβCD for cholesterol can be used not only to remove cholesterol from biological membranes but also to generate cholesterol inclusion complexes that donate cholesterol to the membrane [17]. The molar ratio between cholesterol and cyclodextrin in the complex determines whether it will act as cholesterol acceptor or as cholesterol donor. Cholesterol repletion experiments performed at 15 mM MβCD/cholesterol showed a recovery of both FR and FI of MβCD-treated oocytes, particularly of the FI in which reversibility was close to the control level (Fig. 2A,B). Extrusion of the second PB was also restored (Fig. 3B) suggesting that at this concentration the drug is not toxic for oocytes. Increasing the time of incubation with sperm in the absence of MβCD/cholesterol complexes brought on a recovery of PB extrusion (data not shown). This means that depleted oocytes actually show a delay in the extrusion of the PB. Indeed, there was a time-dependent recovery of the three parameters (FR, FI and PB extrusion). Under our experimental conditions, MβCD-treated oocytes that were not inseminated did not show activation indicating that the drug alone does not reproduce this sperm induced response.

Cholesterol depletion effects induced by MβCD were compared with those of another compound that can bind to cholesterol and disrupt membrane rafts by directly inserting into membranes and sequestering cholesterol into complexes but without removing it (nystatin).

ZP-free ovulated oocytes treated with nystatin survived to the treatment and fertilized. Cholesterol sequestration decreased by about 30% the FI (Fig. 2C) without affecting the FR or the extrusion of the second PB. Control oocytes, maintained in the culture medium supplemented with 5% DMSO, were similarly fertilized than control oocytes maintained in the culture medium only.

### Subcellular Distribution of BODIPY-cholesterol in Mouse Oocytes

**Figure 4:**
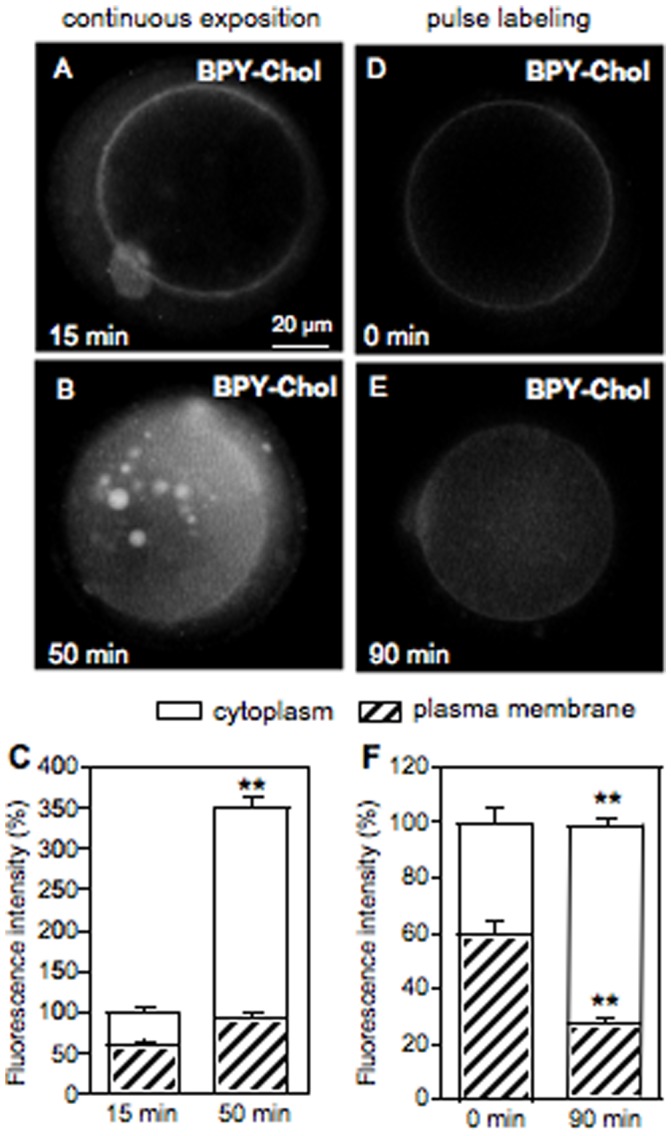
Subcellular localization of BODIPY-Cholesterol in the mouse oocyte. Zona-intact ovulated oocytes were incubated with the fluorescent cholesterol probe for 15 min at 37°C. (A,B) Oocytes continuously incubated with BPY-Chol were imaged at 15 and 50 min. (D,E) Pulse-chase experiment. After incubation, BPY-Chol was washed and followed in time. (C,F) Fluorescence intensity quantified with *ImageJ* software. The bars represent the mean ± SEM of a total of 15 oocytes for continuous exposition experiment and 15 oocytes for pulse-chase experiment. Comparison of mean values for each subcellular compartment over time was performed using Student *t* test. Asterisks denote significant differences (*P*<0.01). Fluorescence of oocytes measured after 90 min was normalized to 100%.

**Figure 5:**
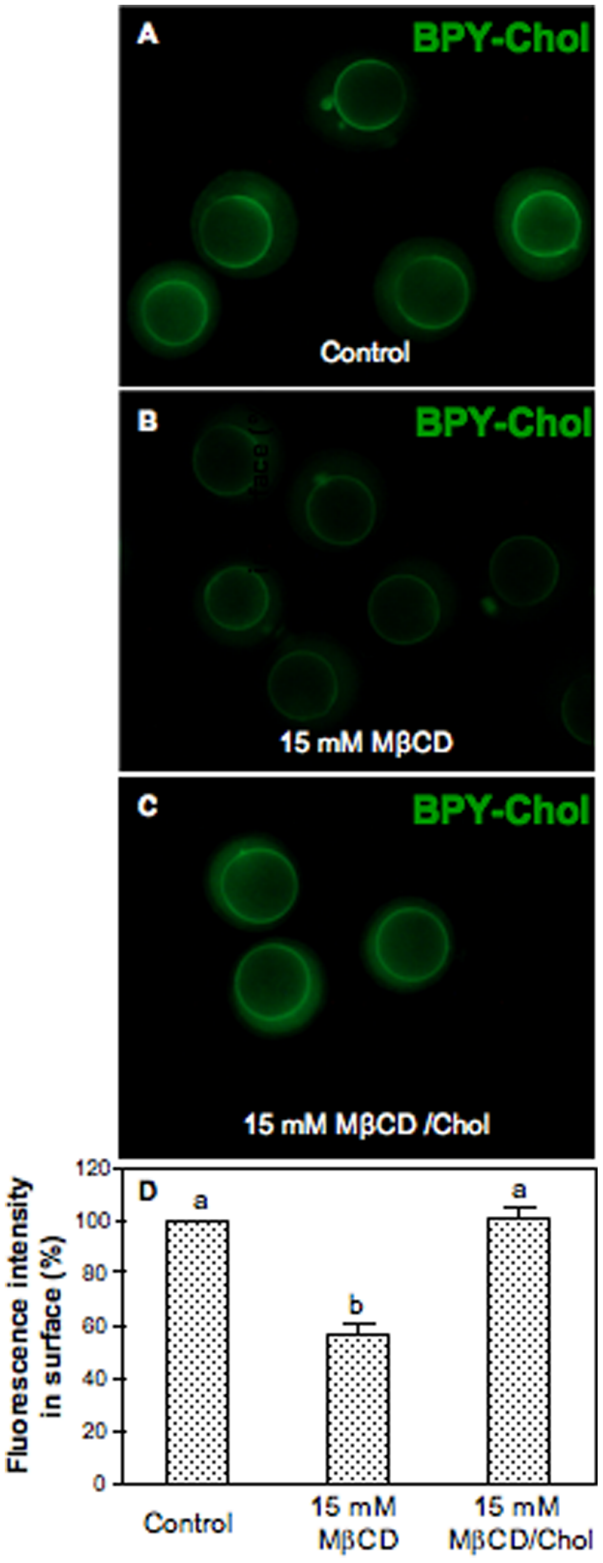
Effect of cholesterol depletion and repletion on oocyte cholesterol content. Zona-intact ovulated oocytes were pretreated with 15 mM MβCD for 30 min at 37°C to remove cholesterol. Cholesterol repletion was carried out incubating MβCD-treated oocytes with MβCD/Chol complexes. After depletion/repletion treatment, oocytes were washed and incubated with BPY-Chol for 15 min at 37°C. (A) Control, (B) depleted and, (C) depleted/repleted oocytes labeled with BPY-Chol. (D) Fluorescence intensity quantified with *ImageJ* software. Bars represent the mean ± SEM of 3 independent experiments from a total of 14 control oocytes, 23 depleted oocytes, and 17 depleted/repleted oocytes. Comparison of mean values was performed using Bonferroni test. Different letters (a-b) denote significant differences (*P*<0.05).

To further investigate the effect of MβCD on oocyte cholesterol and estimate the extent of cholesterol depletion and repletion, we used a new available fluorescent probe, a cholesterol compound with a boron dipyrromethene difluoride moiety referred to as BODIPY-Cholesterol. When ZP-intact oocytes were imaged immediately after a 15 minutes labeling at 37°C with the lipid probe BPY-Chol, prominent labeling of the plasma membrane was observed (Fig. 4A). Continuous exposition to the fluorescent cholesterol for 50 minutes not only labeled plasma membrane but also markedly labeled intracellular membranes (Fig. 4B,C). The increased level of cholesterol incorporation after 50 minutes of incubation also resulted in the accumulation of the fluorescent probe in structures that resemble lipid droplets as judge by their size, shape, distribution and function as storage sites for cholesterol esters and triacylglycerols (Fig. 4B). In addition, as BPY-Chol is highly photostable, we were able to follow it with time-lapse imaging in a pulse-chase experiment in which the remaining lipid probe was removed after 15 minutes of exposition (Fig. 4D,E). Interestingly, total fluorescence after 90 minutes was not similar to that of the time zero condition. Indeed, the absolute value of total fluorescence was twice that at time zero. This increase in total fluorescence is explained by the fact that some BPY-Chol remained available in the perivitelline space even after washings (Fig. 4D) and living oocytes continued to recruit this fluorescent probe. For this reason, to analyze comparable changes in the distribution of cholesterol among subcellular compartments, fluorescence of those oocytes followed after 90 minutes was normalized to 100%. Therefore, with increasing chase time, plasma membrane labeling decreased and intracellular structures became visualized indicating that the fluorescent cholesterol distributed among cell membranes (Fig. 4F). The percentage distribution of the label changed during sterol sequestration, showing more than 75% of the fluorescence inside the oocyte (Fig. 4E,F). The timing of these experiments allowed us to find the time-point (15 minutes) at which the fluorescent cholesterol is highly located at the plasma membrane. Thus, the extent of the cholesterol depletion at the plasma membrane was estimated in MβCD-treated oocytes labeled with BPY-Chol (Fig. 5). Cholesterol-specific fluorescence in the plasma membrane decreased about 40% after MβCD treatment (Fig. 5B,D) compared to BPY-Chol-control oocytes (Fig. 5A). Moreover, after repletion treatment cholesterol was incorporated into mouse oocytes in a reversible manner reaching the level of the control oocytes (Fig. 5C,D).

### Localization of the Raft Marker Lipid GM1 in Living Oocytes

**Figure 6:**
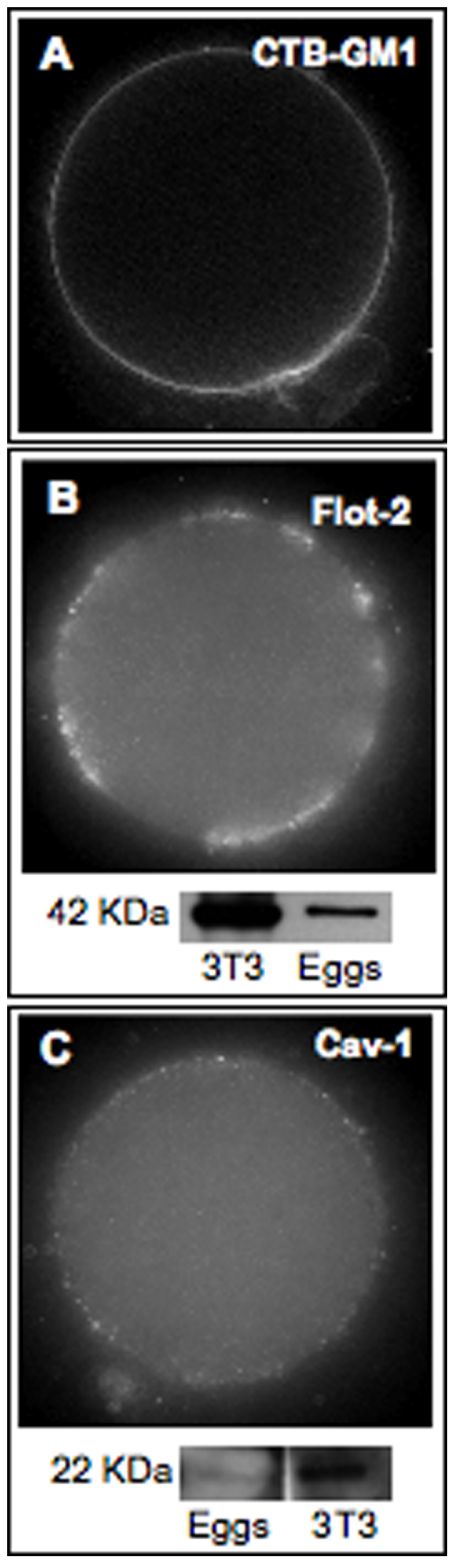
Presence of the raft markers GM1, Flotillin-2 and Caveolin-1 in the mouse oocyte. (A) Plasma membrane localization of the raft marker lipid GM1 assessed by incubation of living oocytes with CTB-AF^488^. (B) Indirect immunofluorescence detection in fixed oocytes and immunoblot detection in whole oocyte lysates of flotillin-2 and (C) caveolin-1. Fluorescence staining was performed in a total of 35 oocytes for GM1, 13 oocytes for flotillin-2 (Flot-2), and 10 oocytes for caveolin-1 (Cav-1). For the Western blots, numbers to the left of each panel indicate the molecular weight of the protein. A total of 120 (Flot-2) and 470 oocytes (Cav-1) were pooled and lysed. 3T3 cell lysates were used as positive controls.

Gangliosides are glycosphingolipids that contain sialic acid in their structure and, in particular, ganglioside GM1 has been extensively used as a marker for raft domains [18], [19]. To confirm the presence of GM1 in the mouse oocyte we used CTB-AF^488^ that recognizes with high affinity the cell surface GM1. Binding of the B subunit to GM1 enables CTB endocytosis. In this respect, live cell imaging at 4°C was crucial for membrane raft staining. Specific binding toxin-GM1 showed a relatively homogeneous distribution of the raft marker lipid exclusively on the oocyte plasma membrane (Fig. 6A).

### Immunodetection of the Raft Marker Proteins, flotillin-2 and caveolin-1

We also evaluated the oocyte localization of the raft proteins flotillin-2, a marker of planar microdomains, and caveolin-1, the structural protein of caveolae by immunofluorescence. As flotillins (flotillin-1 and flotillin-2) localize at the cytoplasmic leaflet of the plasma membrane via acylations and hydrophobic stretches of amino acids, fixation of oocytes was required for indirect labeling. As shown in Figure 6B, we found a strong presence of flotillin-2 on the oocyte plasma membrane, as punctuations along specific enriched areas.

Indirect immunofluorescence staining of caveolin-1 appears as regular punctuations along the oocyte plasma membrane, however at a lesser extent than flotillin-2 (Fig. 6C). Since this is the first report of an immunolocalization of flotillin-2 in oocytes, whatever the species, the corresponding protein expression was confirmed by immunoblot analysis. As the presence of caveolin-1 in the mouse oocyte has never been confirmed by Western blot, it was also evaluated. A single specific band of 42 kDa, the expected molecular weight of flotillin-2 (Fig. 6B), as well as a single specific band of 22 kDa, the expected molecular weight of caveolin-1 (Fig. 6C) were detected in whole oocytes.

### Observation of Caveolae-like Invaginations on Ovulated Mouse Oocytes

**Figure 7:**
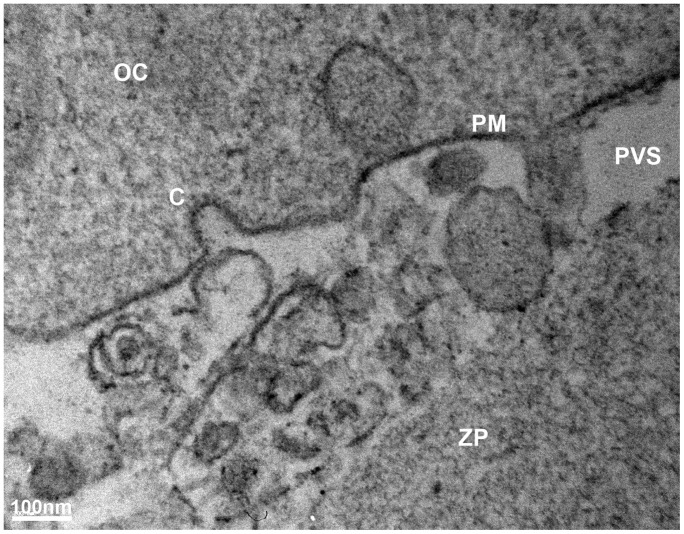
Electron micrographs of caveolae-like microdomains in the mouse oocyte. Ultrastructural plasma membrane with a caveolae-like invagination. OC: Oocyte Cytoplasm; PM: Plasma Membrane; PVS: PeriVitelline Space; C: Caveola; ZP: Zona Pellucida.

Caveolae, are the only membrane microdomains that can be identified morphologically. By transmission electron microscopy, they appear as structures resembling ‘little caves’, which are small flask-like vesicular invaginations of the plasma membrane of 50–100 nm in diameter [20]. According to these criteria, this is the first report that shows characteristic caveolae-like invaginations in the mouse oocyte identified by electron microscopy (Fig. 7).

### Effect of Cholesterol Depletion on Raft- and Non-raft Associated Proteins Src kinase and Cd9 Tetraspanin

**Figure 8:**
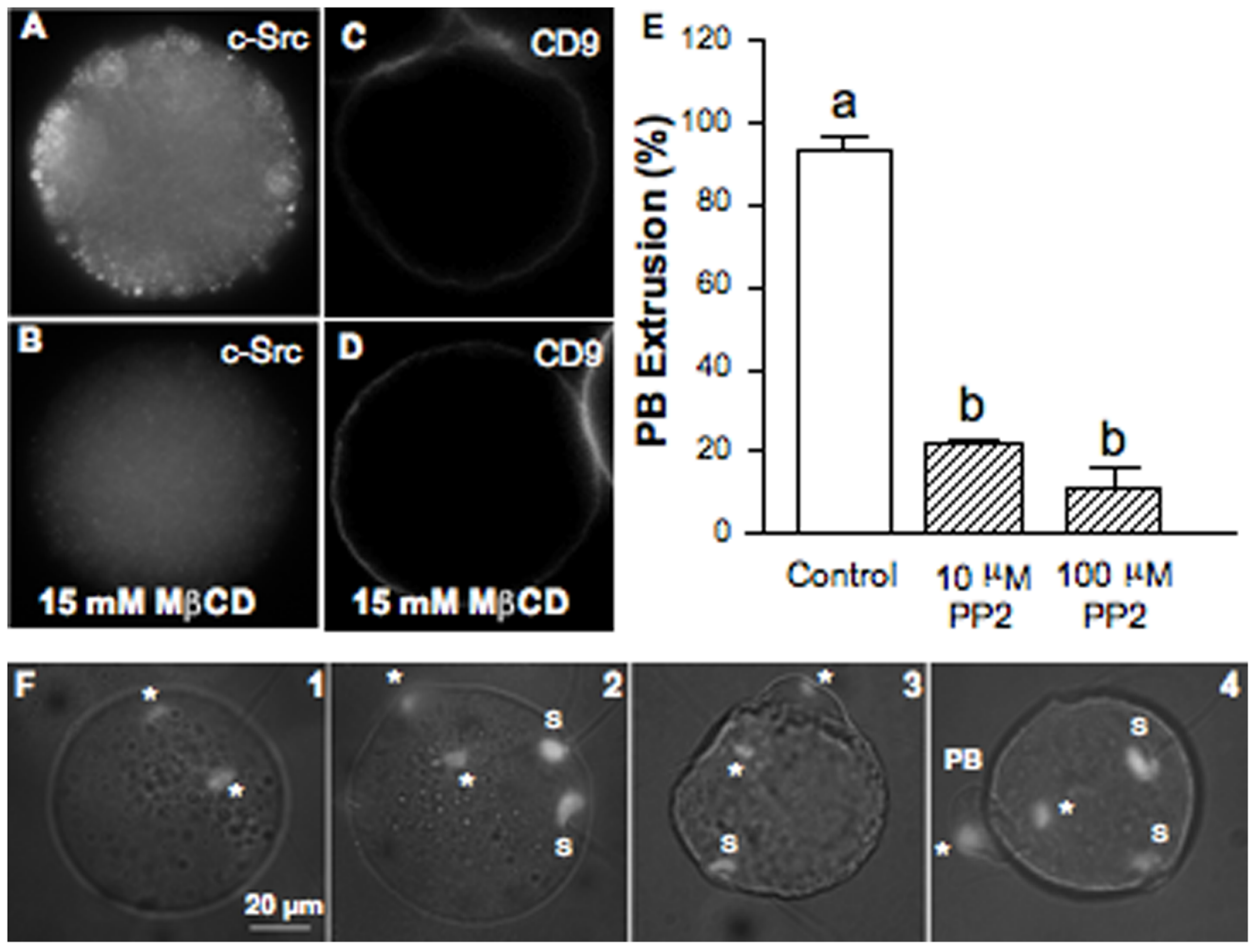
Effect of cholesterol depletion on c-Src and CD9 subcellular localization. Src-family kinase role on second polar body extrusion. (A) Cortex localization of the raft-associated tyrosine kinase c-Srcassessed by indirect immunofluorescence. (B) Cytoplasmic relocation of the c-Src kinase after MβCD treatment. No primary antibody controls were negative. Staining of a total of 14 control oocytes and 6 MβCD-treated oocytes. Within each group, oocytes showed the same staining pattern. (C) Plasma membrane localization of the CD9 tetraspanin, a non-raft protein. (D) CD9 remained at the plasma membrane after MβCD treatment. Staining of a total of 18 control oocytes and 18 MβCD-treated oocytes. In both groups, oocytes showed the same staining pattern. (E) Effect of Src-family kinase inhibition assessed by incubation of oocytes with PP2 on the extrusion of the second polar body (PB). Data represent the mean ± SEM of 3 independent experiments from a total of 77 control oocytes and 85 or 69 oocytes treated with PP2 at 10 or 100 µM, respectively. (F) DAPI-stained images illustrating 1- a blocked telophase, 2- the beginning of the formation of the PB, 3- its almost complete formation, and 4- an extruded PB. Comparison of mean values was performed using Bonferroni test. Different letters (a-b) denote significant differences (*P*<0.05). *: oocyte chromatin; S: sperm decondensed chromatin; PB: Polar Body.

Signaling molecules such as Src family kinases have been shown to be enriched in membrane rafts and are usually used as raft markers. c-Src kinase expression in the mouse oocyte was evaluated by immunofluorescence. In addition, functionality of membrane rafts was assessed by disruption of these microdomains and evaluation of c-Src staining in MβCD-treated oocytes. Labeling of fixed and permeabilized oocytes showed fluorescence punctuations along the cortex of the eggs (Fig. 8A) consistent with the pivotal role of Src kinases as membrane-attached molecular switches that link a variety of extracellular cues to critical intracellular signaling pathways. Cholesterol removal disturbed membrane localization of c-Src significantly decreasing fluorescence intensity at the cortex of the cell (Fig. 8B).

On the other hand, to check that MβCD-induced effects on oocyte fertilization are a direct consequence of raft-cholesterol depletion without affecting non-raft cholesterol function, expression level of a non-raft protein (Cd9) in MβCD-treated oocytes was evaluated. The incubation of oocytes with MβCD did not modify the expression of Cd9 at the oocyte plasma membrane (Fig. 8C,D). MβCD seems selectively alter the expression of proteins found in cholesterol rich rafts without affecting non-raft associated proteins. In agreement with the reported effects on Src localization, this also means that, at least under our experimental conditions, the removal of cholesterol by MβCD has mainly affected oocyte membrane rafts.

### Effect of Src Kinase Inhibition on PB Extrusion

Similarly to cholesterol depletion, MII oocyte treatment with PP2, a potent inhibitor of Src family members, significantly prevented PB extrusion (Fig. 8E). No PB were visible in 71.7% of the oocytes maintained in contact with spermatozoa during 1 hour when PP2 was used at 10 µM, a more important inhibition being observed when PP2 was used at 100 µM, since less than 12% of the fertilized oocytes extruded the PB. This effect is illustrated by DAPI-stained images (Fig. 8F). In most of the cases, segregation of oocyte chromatids was arrested within the ooplasm (Fig. 8F1), but sometimes the beginning of the formation of the PB (Fig. 8F2), or its almost complete, or totally complete formation were observed (Fig. 8F3 and 4, respectively). However, this arrest was actually a delay since after 2 hours of recovery after insemination the rate of oocytes with extruded PB was similar to that of the control (data not shown).

## Discussion

Working on mammalian gametes adhesion/fusion, it was worthwhile to highlight the involvement of membrane rafts, which permit membrane sub-compartmentalization, regulating membrane bioactivity [8] since the oocyte membrane reorganization is a crucial event at this moment (for review [7]). In rafts, gangliosides are important in organizing the fine structure of cellular membranes. Important biological events are likely to be affected such as the dynamic control of the shape of specialized plasma membrane areas and of the intracellular organelles, the in- and outward budding and fusion of membrane vesicles, the physical and functional coupling of the outer and the inner plasma membrane leaflets, involved in the transduction of signals across the membrane. Our data confirm that the mouse membrane oocyte is rich in rafts according to the presence of the glycolipid GM1 all along the membrane of living oocytes, as previously demonstrated on fixed mouse oocytes and embryos as well as an evident enrichment at the cleavage furrow during cytokinesis observed short after fertilization [10] or more recently in living mouse embryos [11]. By contrast, it has been reported that biotinylated-cholera toxin accumulate in the perivitelline space in unfertilized mouse eggs, whereas only a small amount of GM1 was detected at the interfaces in compacted 8-cell stage living embryos [11]. Our experimental conditions to visualize GM1 in living oocytes could be an alternative starting point to further investigate GM1 localization in mouse preimplantation embryos.

Cholesterol depletion by MβCD induced decrease of fertilization rate and index. It is important to underline that the MβCD effect is non cytotoxic, since the repletion in cholesterol permitted to partly recover the FR and completely recover the FI. This recovery occurred also in the absence of added cholesterol but with delay suggesting that it could be due to cholesterol synthesis in the oocyte as already shown for MDCK II cells [21]. Interestingly, cell death induced by MβCD after long time incubation and with increasing concentrations of the drug, occurred as a non-apoptotic mechanism in several cell types (NR8383 cells, A549 cells and Jurkat cells) [22]. In our study, no DNA fragmentation was observed in MβCD-treated oocytes after staining with the DNA-binding fluorescent dye, DAPI. Therefore, it is also likely that other mechanism different from apoptosis could be operating in those oocytes that did not resist treatment with MβCD.

Regarding non-specific effects of MβCD on non-cholesterol membrane components, it is important to take into account that MβCD may interact with hydrophobic amino acids and phospholipids due to the hydrophobic character of its pocket. Several studies have shown membrane release of certain proteins and phospholipids after βCD treatment [22], [23]. However, until now there are no sufficient systematic studies about the interaction of βCDs with cell surface proteins or phospholipids to predict the effect of these compounds on cells in specific situations. Useful control strategies may help to verify that the observed effects are due specifically to cyclodextrin-induced changes in cellular cholesterol. In this respect, cholesterol repletion solely may not restore membrane functionality if an involved protein/phospholipid was severely removed by MβCD treatment. Thus, under our experimental conditions, mainly cholesterol may account for the ability of mouse oocytes to recover their fertilization competence. Importantly, analysis of the expression of raft (c-Src) and non-raft (Cd9) proteins indicated that MβCD mainly affected proteins associated to cholesterol rich rafts without affecting non-raft associated proteins.

Nystatin partially supported these findings by decreasing the number of decondensed sperm per oocyte but without affecting the FR and the extrusion of the second PB. Sequestering cholesterol with filipin or nystatin or modification of membrane cholesterol by its enzymatic degradation with cholesterol oxidase [24] or by serum starvation [25], are methods used to compare the effects of cholesterol depletion induced by MβCD. However, these methods, introduce additional factors difficult to quantify, such as changes in the local concentration of cholesterol or a build-up of products of cholesterol degradation [17]. Conversely, in MβCD-treated oocytes it was possible to correlate functional effects to cholesterol levels and restored this condition by cholesterol repletion. Marginal effects of nystatin might be also explained by the poor aqueous solubility and stability of this polyene anifungal agent.

What does appear clear is that the disruption of the basic structure of cell membranes composed of two apposing leaflets, where sphingolipids and cholesterol assemble, affects the process of fertilization. This result has recently been observed [26], however without dose response curve. The authors have observed a decrease in the fertilization index of mouse oocytes after treatment with cyclodextrin for an unspecified time of incubation. On the other hand, in non-mammalian species, it has been demonstrated that treatment with the cholesterol depleting drug, MβCD, inhibited amphibian oocyte maturation by disturbing the integrity of membrane rafts [27].

Our work is the first in which the fluorescent probe BODIPY-Chol was used to investigate plasma membrane microdomains in mouse living oocytes. This novel approach permitted to identify the time-point at which the fluorescent cholesterol is mainly located at the plasma membrane enabling the estimation of the extent of the cholesterol depletion. In addition, increased lipid probe internalization resulted in its accumulation in cytoplasmic structures that resemble lipid droplets. The main important functions of the lipid droplets are to regulate the intracellular level of free fatty acids and free cholesterol, to be the site of synthesis and metabolism of a wide range of lipids, to release fatty acids preferentially used for many physiological functions over fatty acids taken up from the extracellular milieu or synthesized de novo in the cell and finally to provide a binding surface for proteins [28]. At present, lipid droplets are under intensive study due to the increasing recognition that they have significant roles in many aspects of health and disease [29]. In particular, a metabolic role for lipid metabolism during porcine oocyte maturation has been demonstrated by using inhibitors of fatty acid β-oxidation leading to developmental failure post-fertilization [30]. Just as the induction of perilipin-2, a lipid droplet protein in mouse oocytes concurrent with dynamic reorganization of lipid droplets suggests marked changes in lipid utilization during oocyte maturation [31].

Our data also show that two kinds of membrane microdomains coexist in mammalian oocytes: flat lipid rafts revealed by the presence of flotillin-2 and flask-shaped plasma membrane invaginations or caveolae-like microdomains revealed by the presence of caveolin-1 and confirmed by transmission electron microscopy. Flotillin-2 is considered a better marker protein for lipid rafts than flotillin-1 because besides its dual palmitoylation is irreversibly myristoylated being the more immobile of the two [32]. On the other hand, flotillin-1 expression seems to be more restricted at the cellular level [32]. Flotillins are also considered to be scaffolding proteins of lipid microdomains [33]. This protein family has never been observed in the oocyte before, whatever the species, but flotillin-1 has been observed in the porcine cumulus cells evolving throughout oocyte maturation [34]. It is also interesting to note that flotillins promote the local co-assembly of specific GPI-anchored proteins on the cell surface and allow interaction with signal transduction molecules, including the Src-family protein tyrosine kinases (PTK) [13].

Caveolin-1 is an ubiquitously expressed integral membrane protein, essential for the formation of so-called caveolae, small invaginations of the plasma membrane involved in major physiological functions of the mammalian cell [35]. Among caveolins, caveolin-1 is the true protein marker of caveolae. Caveolin-2 colocalizes with caveolin-1 in caveolae but requires caveolin-1 for proper membrane localization and caveolin-3 has greater protein-sequence similarity to caveolin-1, but it is expressed mainly in muscle cells [20]. By contrast to flotillins, caveolins have been found to be expressed in oocytes of different species such as the nematodes *Caenorhabditis elegans* [36], [37] and *Trichinella spiralis* [38], the amphibian *Rhinella arenarum* [27], [39] and human [40]. More recently it has also been observed in the cytoplasm of mouse oocytes on slides of ovary, however without any precision on the follicular size or the oocyte meiotic status [41]. In line with this, we confirmed for the first time by electron microscopy the presence of caveolae-like microdomains at the ultrastructural level of the mouse oocyte membrane. In contrast, flat rafts do not have the caveolae-like morphology and have less than 50 nm in size. Caveolae are also distinguishable from clathrin-coated pits (>100 nm) by their minor size and the lack of the clathrin lattice-like coat [42]. In *Rhinella arenarum* oocyte, the presence of raft markers and the finding of signaling molecules from the MAPK cascade functionally associated to oocyte membranes suggest that this caveolae-rich fraction efficiently recreates, in part, maturation signaling [43].

Regarding signaling pathways, we also observed the presence of the c-Src kinase along the oocyte cortex. The drastic disturbance of its localization after microdomains disruption by cholesterol removal highlights its dependence on intact membrane rafts. It was already known that mammalian eggs express Fyn, Yes and in some cases, Src [44], [45] but these kinases have not been described as required for the unique sperm-induced calcium oscillations [46], [47], [48], which trigger egg activation in mammals [49]. Numerous studies involving chemical inhibitors, dominant negative fusion proteins and exogenous recombinant kinases have demonstrated that PTKs including Src-family PTKs, play an important role in activation of eggs from non-mammalian species. These species typically exhibit a rapid activation of Src-family PTKs which may play a role in sperm-egg fusion [14], [50], [51], [52] and are required for the rapid, high amplitude calcium transient that triggers egg activation [53], [54]. Our data verified that Src-family kinases are involved in the completion of mouse oocyte meiosis since its inhibition by the specific inhibitor PP2 before fertilization significantly delays the extrusion of the second PB as does the inhibition of Fyn, a member of the Src family proteins, on rat oocytes [55]. PP2 also significantly reduced post-insemination levels of PB formation in the marine protostome worm *Cerebratulus* [56]. Furthermore our data revealed similarities with the effect shown after MβCD-mediated cholesterol depletion on PB extrusion suggesting the assembling of membrane-related proteins such as c-Src in signaling complexes compatible with the role of membrane rafts. Interestingly, the contractile ring in sea urchin embryos has been associated with GM1 and cholesterol-rich microdomains that are characterized by intense PTK signalling [57], and it is likely that a similar mechanism is employed during PB extrusion. Moreover, GM1 associates with complexes formed by uroplakin proteins and contributes to Src-dependent activation of *Xenopus laevis* eggs [58]. It is also known that caveolin scaffolding domain contains motifs that bind signal molecules such as Src [59].

As discussed above, two oocyte proteins are essential to gametes adhesion and fusion, the tetraspanin Cd9 and at least one GPI-anchored protein. Both proteins associate to specific microdomains on cell plasma membrane, TEM for the first one and non-invaginated rafts for the second. Whereas tetraspanins are demonstrated to belong to cholesterol-depletion-resistant membrane microdomains [60], a physical and functional link between tetraspanins and cholesterol has also been shown [61]. Links between TEM and membrane rafts have first been considered as impossible, however it has been recently demonstrated that they can relate, in particular in immune cell signaling, malignant disease and HIV-assembly [62], [63], [64], [65]. The following data can at least partly explain the contradictory observations [66]: in contrast to *Plasmodium* infection, the association of Cd81 with TEM is not essential for the early steps of HCV life cycle, indicating that the same molecule can work by different mechanisms. In fact, cholesterol depletion inhibited HCV infection and reduced total cell surface expression of Cd81, without affecting TEM-associated Cd81 levels. Finally, gangliosides such as GM2 and GM3, which associate either independently or in complex with tetraspanins, in particular Cd9, promote its interaction with other proteins leading to its cell function [67], [68]. Another example demonstrates links between tetraspanins and membrane rafts: the disruption of the membrane with MβCD dissociates the EGFR/GM3/caveolin-1/CD82/PKC-alpha complex and prevents the inhibitory effect of PKC-alpha on EGFR phosphorylation, suggesting that caveolin-1, CD82, and the ganglioside interact with EGFR and PKC-alpha within intact cholesterol-enriched membrane microdomains [69].

A protocol for studying the molecular mechanism of egg fertilization has been published few years ago, using cell-free extracts and membrane/lipid rafts prepared from unfertilized, metaphase II-arrested *Xenopus* eggs [70]. It has permit to reconstitute a series of signal transduction events associated with egg fertilization, such as sperm-egg membrane interaction, activation of Src tyrosine kinase and phospholipase C gamma, production of inositol trisphosphate, transient calcium release, and cell cycle transition. It would be very interesting to develop this type of reconstitutional system in the mammalian oocyte but it still remains difficult due to the limitations on the number of oocytes required. Recently, by using the force measurement technique to quantify subtle local changes in membrane adhesion, we have discriminated different types of adhesive interactions between sperm and egg plasma membrane during the fertilization process. Cd9 tetraspanin is responsible for strong adhesion generating fusion competent sites [71]. However, Cd9 tetraspanin essential in mammalian sperm-egg membrane adhesion/fusion does not contain a fusion peptide. It remains to verify by our biophysical approach whether GPI-anchored proteins, one or more GPI-anchored egg surface proteins being essential for sperm-egg binding and fusion [6], [72], which role they play in the adhesion step. Moreover, flotillin proteins are widely clustered at contact sites between cells [73] and GPI-anchored proteins are strongly associated with flotillin-enriched lipid rafts with lipids as cue components in cell-cell fusion as demonstrated by our data in gametes interaction. Previously, we have proposed a model [7] in which oocyte membrane rafts migrate to contact/fusion sites favoring clustering and protein-protein interactions. Tetraspanins and GPI-anchored proteins cluster in the membrane, and both associate with integrins. As GPI-anchored proteins, several integrins have also been found associated to membrane rafts [74], [75]. Plasma membrane cholesterol is therefore a key player in the different stages of oocyte fertilization, i.e. adhesion and fusion. Interestingly, it has been recently shown that cholesterol mediates membrane curvature during fusion events [76]. Cholesterol affected the conformation of the glycoprotein gp41 fusion domain of the HIV-1 virus promoting a beta-sheet structure over alpha-helix [77], [78] and regulated its membrane penetration depth and occupied surface area in model systems [76]. For low cholesterol concentrations in the opposing membrane, the protein domain embeds with a large expansion of area at the level of head groups leading to a significant positive curvature in the lipid bilayer that is essential at the initial stage of the fusion pore formation. In contrast, with a higher cholesterol concentration, the fusion peptide expands the hydrophobic and hydrophilic regions almost equally with a milder effect on the overall curvature of the host membrane representing later stages of the fusion process. Thus, the membrane can bend back-and-forth simply by regulating the local concentration of cholesterol at the point of contact with membrane-bending sequences [76].

Recently, a new model based on myotube formation proposed the involvement of lipid rafts, adhesion proteins and actin rearrangement in cell fusion [79]. In this model, membrane rafts first recruit adhesion molecules and align with opposing membranes to finally disperse and expose a highly fluidic bilayer leading to direct contact and the formation of fusion pores by actin polymerization force. Despite the diversity of fusion events, recent advances in the field start to reveal common mechanisms also in gamete interaction [80], [81]. The efflux of cholesterol that occurs during sperm capacitation favoring an overall more fluid plasma membrane and thus making it more fusogenic supports this hypothesis. Conversely, the acrosome reaction primes sperm for fusion facilitating relocation of sperm-egg fusion proteins, such as Izumo and flotillin-2, into the plasma membrane [82], [83].

Based on all these observations, it seems likely that also after sperm-egg lipid bilayer mixing and expansion of fusion pores, once again cholesterol concentrates at the fusion site to bend the membrane back. In addition, due to the large difference in size between an egg and a sperm, it may be mainly the sperm membrane, which undergoes greater positive curvature to adapt to a more ordered oocyte membrane at the moment of fusion. Note that the contribution of the sperm membrane in terms of lipid mass is minor compared to that of the oocyte membrane which also includes a microvillar region. It remains to be established whether a bending sequence is acting to produce this curvature at the sperm plasma membrane. Thus, dynamic successions of membrane raft clustering and dispersion may account for gamete adhesion/fusion with these organizing platforms acting either prior to oocyte-sperm membrane fusion as well as in the final stages of the fusion process. Experiments showing depletion of membrane raft cholesterol provide a straightforward answer to this phenomenon. Here, we show evidence that membrane raft integrity is necessary to efficiently accomplish fertilization in the mouse oocyte. A lipidomic approach would be very interesting to describe the lipid composition of gametes membrane in order to study the degree of contribution of each component in female and male gametes adhesion and fusion.
